# Immune dysregulation as a driver of bronchiolitis obliterans

**DOI:** 10.3389/fimmu.2024.1455009

**Published:** 2024-12-17

**Authors:** Kuimiao Deng, Gen Lu

**Affiliations:** Department of Respiration, Guangzhou Women and Children’s Medical Centre, Guangzhou Medical University, Guangzhou, Guangdong, China

**Keywords:** bronchiolitis obliterans, bronchiolitis obliterans syndrome, immune dysregulation, immune cells, fibrosis

## Abstract

Bronchiolitis obliterans (BO) is a disease characterized by airway obstruction and fibrosis that can occur in all age groups. Bronchiolitis obliterans syndrome (BOS) is a clinical manifestation of BO in patients who have undergone lung transplantation or hematopoietic stem cell transplantation. Persistent inflammation and fibrosis of small airways make the disease irreversible, eventually leading to lung failure. The pathogenesis of BO is not entirely clear, but immune disorders are commonly involved, with various immune cells playing complex roles in different BO subtypes. Accordingly, the US Food and Drug Administration (FDA) has recently approved several new drugs that can alleviate chronic graft-versus-host disease (cGVHD) by regulating the function of immune cells, some of which have efficacy specifically with cGVHD-BOS. In this review, we will discuss the roles of different immune cells in BO/BOS, and introduce the latest drugs targeting various immune cells as the main target. This study emphasizes that immune dysfunction is an important driving factor in its pathophysiology. A better understanding of the role of the immune system in BO will enable the development of targeted immunotherapies to effectively delay or even reverse this condition.

## 1 Introduction

Bronchiolitis obliterans (BO) is a chronic, irreversible pulmonary disease characterized by small airway obstruction and/or occlusion and peripheral and distal bronchiolar fibrosis (1). Typical signs and symptoms include dyspnea, wheezing, and hypoxemia. However, the patient may be asymptomatic, and BO can be diagnosed at an early stage by pulmonary function test (PFT) and computed tomography (CT) (2). The presence of obstructive airflow patterns in PFT and air trapping detected by CT during exhalation are characteristic indicators of BO (3). BO can occur in all ages, but its etiology differs in children and adults. Bronchiolitis obliterans syndrome (BOS) is a clinical manifestation of BO in patients who have undergone lung transplantation or hematopoietic stem cell transplantation (HSCT) (3). BO occurs in different diseases and is thus variously categorized under different medical terms. Chronic lung allograft dysfunction (CLAD)-BOS and chronic graft-versus-host disease (cGVHD)-BOS are the most common etiologies in adults (4), whereas post-infection-BO (PIBO) most commonly affects children (5).

PIBO is one of the rare complications of severe lower respiratory tract infections (6). Although PIBO can occur in patients of any age, it is more common in children, most often in association with adenovirus infection (7–10). However, other viruses (e.g., influenza, parainfluenza, respiratory syncytial virus, human metapneumovirus, human immunodeficiency virus-1, measles, cytomegalovirus, Sars-Cov-2) and bacteria (*Mycoplasma pneumoniae*, *Legionella pneumophila*, *Bordetella pertussis*) can also cause PIBO in the event of severe lower respiratory tract infection (11, 12). Based on histopathological features, BO can be categorized into two types: 1) proliferative BO, characterized by granulation tissue polyps obstructing the small airway, and 2) contractile BO, marked by peribronchiolar fibrosis resulting in varying degrees of lumenal constriction. As the disease progresses, the histological features of PIBO primarily exhibit a contractile pattern, accompanied by variable degrees of dilatation and airway obstruction (13). The pathological manifestations of patients with PIBO vary greatly, and the involvement of bronchioles is heterogeneous. Other signs of persistent airway disease in PIBO include bronchiolitis, mucus deposition, macrophage aggregation, and bronchiole distortion and expansion (13–15).

Lung transplantation is considered to be the best treatment option for patients with end-stage lung disease (16). CLAD is an umbrella term used to define a persistent (≥3 weeks) decline in pulmonary function (forced expiratory volume in 1 second (FEV 1), with or without a decline in forced vital capacity) of ≥10% from baseline (17). CLAD is the main cause of death one year after transplantation, and BOS is the most common CLAD phenotype. In the survival of lung transplant recipients, the prevalence rate of CLAD-BOS is 50% after 5 years and 76% after 10 years (16). From the perspective of histopathology, BOS is characterized by the accumulation of extracellular matrix under the mucosa, partial destruction of the original smooth muscle layer, or myocyte proliferation, and ultimately complete airway obstruction (3). Antibodies play an important role in the occurrence and development of CLAD-BOS. Graft-reactive antibodies can induce the activation of the complement system and the degradation of lung tissue, leading to the formation of CLAD-BOS (18).

BOS is a rare complication of allogeneic HSCT, characterized by fixed airflow obstruction after allogeneic HSCT. The incidence of BOS in allogeneic HSCT recipients is about 2-3%, but it can reach 6% in patients with cGVHD (19, 20). The early processes leading to BOS in cGVHD are different from those in CLAD, but the final histological changes are relatively similar (21). However, the etiology of BOS is still unknown. Allogeneic recognition of lung antigens may be the cause of this disease, and BOS involves allogeneic immunity, namely HSCT graft-versus-host (lung) disease. In fact, in cGVHD, lung epithelium may be a target of donor cytotoxic T cells, supporting the hypothesis that BOS is the manifestation of cGVHD in the lungs (22).

This paper summarizes research on patients with BO and animal models and reviews the roles of various immune cells, including macrophages, neutrophils, eosinophils, natural killer (NK) cells, B lymphocytes, and T lymphocytes, on BO onset and progression. The most common cause of PIBO is adenovirus infection. Castleman et al. (23) induced beagle dogs with adenovirus to establish a BO model that adequately reflects the pathophysiological process of BO formation caused by viral infection. However, owing to the considerable costs associated with large animal breeding and experimentation, the model is hindered from widespread adoption. Rodent models are therefore more commonly used, due to lower costs, simpler procedures, and easier reproducibility. However, because respiratory bronchioles are absent or rare in rodents, such studies cannot sufficiently reflect the complexity of clinical BO (24, 25). Hence, the relevance of virus-induced BO rodent models has yet to be validated. For non-transplantation models, diacetyl (26), nitric acid (27), chlorine (28), sulfur mustard (29), and papaverine (30) are currently used more frequently. Still, although these animal models can develop airway obstruction and even fibrosis, they cannot accurately reflect the pathophysiological process of BO. In turn, human research on BO relies on tissue specimens, but histological confirmation is often difficult due to insufficient diagnostic sensitivity of bronchoscopic biopsy specimens. Despite these caveats, animal models that effectively simulate human pathophysiological changes often provide detailed mechanistic insights, which are challenging to obtain in human research.

## 2 The role and mechanisms of immune cells in bronchiolitis obliterans

### 2.1 Monocytes and macrophages

**Figure 1:**
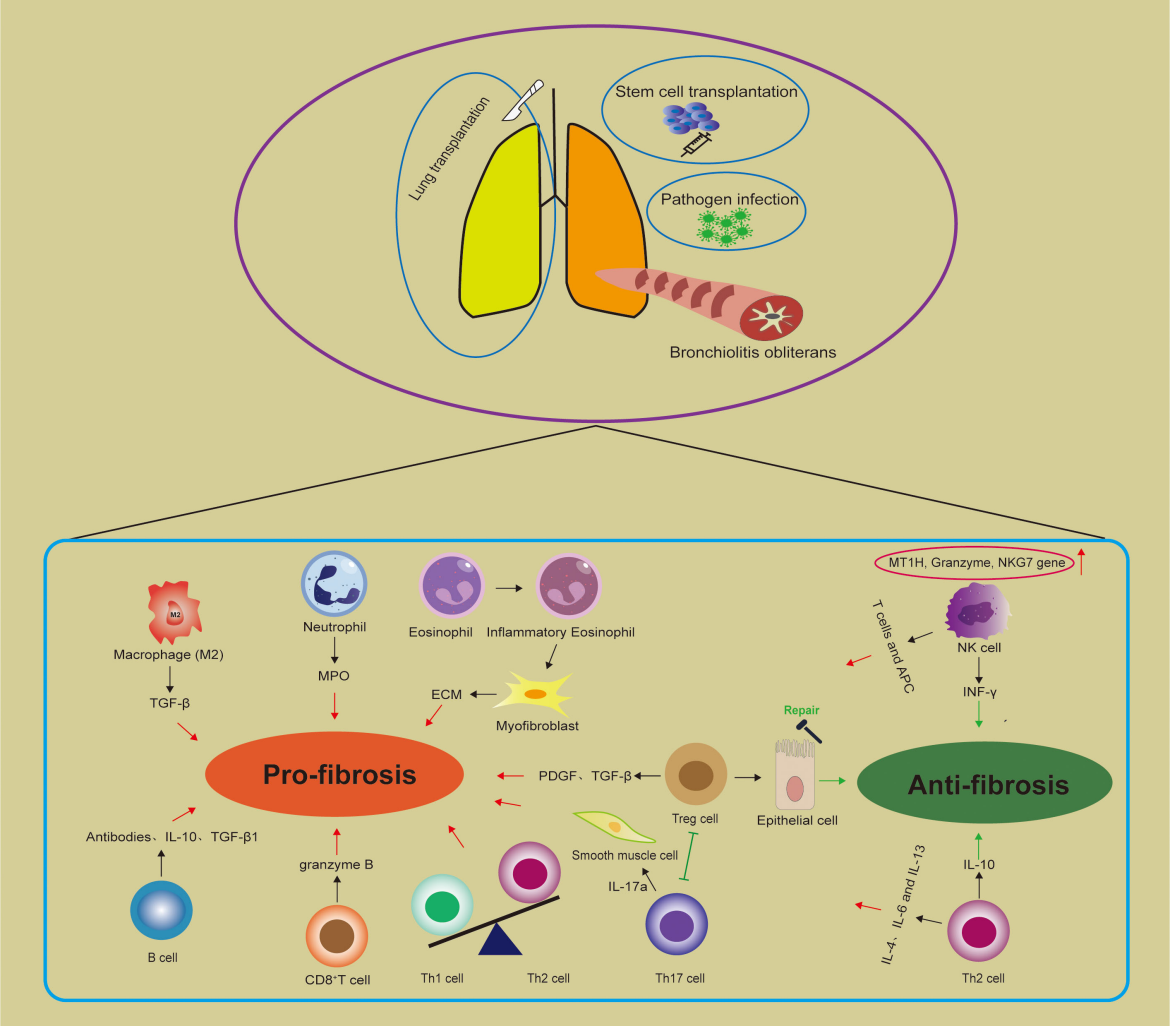
Immune cells in bronchiolitis obliterans (BO). There are three main entities of BO: post infection BO; BO after stem cell transplantation and BO after lung transplantation. Macrophages, neutrophils, eosinophils, Th1 and Th17 cells may contribute to disease progression, while Th2, NK, and Tregs seem to have controversial roles. The balance between Tregs and Th17 cells is implicated in pulmonary fibrosis. MPO, Myeloperoxidase; ECM, Extracellular matrix; Treg cell, Regulatory T cell; NK cell, Natural killer cell; APC, Antigen-presenting cells; MT1H, Metallothionein-1H; NKG7, Natural killer cell granule protein 7.

Monocytes and macrophages are multifunctional immune cells that exist or infiltrate tissues and crucially influence innate immunity, normal tissue development, homeostasis, and repair of damaged tissues (31). Macrophages are involved in various conditions and diseases, such as inflammation, tumors, and autoimmune disorders, and play essential roles in eliminating pathogens and regulating immune function (32, 33). Alveolar macrophages (AM) serve as the primary defenders for the airways and alveoli against pathogens, while pulmonary interstitial macrophages act as vital gatekeepers for the vascular system and pulmonary interstitium (34) (**Figure 1**).

Macrophages play a major role in the occurrence and development of BO. Duecker et al. (35) found that the percentage of macrophages in the sputum of patients with PIBO was significantly lower than that of healthy controls. Similarly, Palmer et al. (36) found that on day 1 post-induction, AM numbers in an animal model of BO were significantly higher than in the control group, while on days 3 and 7, AM abundance decreased slightly. O’Koren et al. (37) observed that AM levels rose during the initial phase and declined during the subsequent phase. In this model, infiltration of inflammatory cells, particularly monocytes and AM, preceded the development of intraluminal lesions, which manifested typically after 7 days of chlorine exposure. Meanwhile, suggesting the potential value of anti-TNF-α therapy in CLAD-BOS, it was further reported that classically activated macrophages adjacent to bronchial epithelial cells of lung transplant patients show increased secretion of TNF-α and IL-1β, in parallel with significantly enhanced epithelial-mesenchymal transition (EMT) driven by transforming growth factor beta-1 (TGF-β1) (38). This indicates that anti-TNF-α can inhibit macrophages driven EMT function and thus treat BOS.

Macrophage dysfunction appears to be involved in the pathogenesis of BOS after lung transplantation, and potential therapeutic targets might be derived from the underlying mechanisms (39). It is controversial whether the main source of AM after transplantation comes from the donor or the recipient. Nayak et al. (40) demonstrated that >94% of AM in the bronchoalveolar lavage fluid (BALF) of human lung transplant recipients are sourced from the donor, being the predominant macrophage subset for at least 2 to 3 years after transplantation (41). The inflammatory cascade reaction caused by donor-derived AM leads to the injury of airway epithelium, which eventually leads to the obstructive airway disease of transplanted lung. However, Snyder et al. (42) found that about 2 years post-transplantation, the proportion of donor macrophages in lung recipients was only about 10.5%. Similarly, after HSCT, the majority of infiltrating AM appears to originate from the donor’s hematopoietic cells (43). Thus, there are conflicting data on the proportion of donor-derived AM in lung transplant recipients.

Based on the activation state and function of macrophages, these cells can be categorized into an M1 phenotype (classically activated macrophages) and an M2 phenotype (alternatively activated macrophages) (44). M1 macrophages are primarily involved in immune responses such as microbial killing and anti-tumor effects, while M2 macrophages are primarily involved in immune suppression and tissue repair. Both macrophage types play essential roles in the immune system, maintaining a dynamic equilibrium. Although the classification of macrophage phenotypes into a M1 and M2 category is oversimplified, the extreme heterogeneity of response to stimuli highlights the need for a better understanding of the function of macrophages in human pathology. The AM observed in the early phase of cGVHD-BOS are positive for CD68 and inducible nitric oxide synthase and negative for CD163 and CD206, suggesting an M1 phenotype. This suggests that donor-derived M1-AM may contribute to the pathogenesis of the early phase of cGVHD-BOS, while AM exhibiting M2 polarization potentially participate in fibrosis in the late phase (43).

During the formation of fibrotic scars, various cells secrete platelet-derived growth factor (PDGF) in response to injury, and many pro-inflammatory cytokines facilitate their mitogenic effects through the autocrine secretion of PDGF. Through immunohistochemical and *in situ* hybridization studies on tissue sections and BALF cells of patients with CLAD-BOS, it was found that AM are one of the cell sources of PDGF (45). TGF-β also plays a significant role in pulmonary fibrosis. During the acute complications of lung transplantation, AM are initially activated, leading to excessive secretion of interleukin-6 (IL-6), which mediates tissue repair (46). High airway TGF-β activity can enhance the expression of chemokine ligand 2 (CCL2) in AM, resulting in the development of BO mouse model driven by CCR2^+^ monocytes (47). It can thus be questioned whether the severity of BO rat model can be reduced by inhibiting the number and activation of AM. It was shown that macrophage depletion with GdCl_3_ significantly reduced the development of occlusive airway disease in experimental heterotopic tracheal allografts, resulting in a decrease in PDGF mRNA expression (48). In turn, inhibition of macrophage migration inhibitory factor (MIF) significantly prevented tracheal epithelial and luminal occlusions caused by fibrosis, reflecting blockade of MIF-related adverse immune reactions associated with allograft rejection (49).

Interestingly, Alexander et al. (50) reported that colony-stimulating factor 1 (CSF-1)-dependent donor derived macrophages mediate the occurrence of cGVHD in mouse models. Axatilimab is a hinge-stabilized IgG4κ antibody targeting the CSF-1 receptor, which is the first cGVHD therapeutic drug targeting disease-related macrophages (51). Among patients receiving approved doses of 0.3mg/kg niktimvo every two weeks (N=79), 75% of patients achieved an overall response rate (ORR) within the first six months of treatment. The pulmonary response rate was 50%, with 16 patients showing complete response (52). In 2024, the US Food and Drug Administration (FDA) has recently granted axatilimab orphan drug designation for patients with cGVHD and idiopathic pulmonary fibrosis.

### 2.2 Neutrophils

During pulmonary infections, neutrophils are recruited to fend off further immune cell recruitment by engulfing necrotic cells. Concurrently, they facilitate tissue growth and neovascularization by releasing cytokines and synthesizing granule enzymes, playing an important role in tissue breakdown and repair (53). Neutrophils are persistently activated at sites of chronic inflammation, fueling the inflammatory process through the release of proteases, the formation of neutrophil extracellular traps (NETs), and the activation of other immune cells (54). As key players in chronic respiratory disorders, neutrophils are integral to the progression of conditions such as chronic obstructive pulmonary disease (COPD), asthma, and pulmonary fibrosis (55).

The characteristic airway fibrosis in BO is due to the inability of the airway epithelium to repair normally after injury (1). In this context, a large number of T cells and neutrophils functionally replace epithelial cells, followed by matrix degradation, collagen deposition, and fibroblast stimulation, ultimately leading to airway fibrosis (6). It was reported that patients with BO/BOS showed a significant increase in the percentage of neutrophils in BALF, sputum, and on histopathology (56–59).

Due to neutrophils’ capacity to induce ongoing airway remodeling and inflammation, their recruitment and activation may cause harm to the lung tissue of patients with CLAD-BOS (60, 61). Azithromycin can significantly reduce the number of airway neutrophils in patients with CLAD-BOS, which may be one of the mechanisms behind its therapeutic effects (62). The early increase of neutrophils in BALF has a predictive effect on the occurrence of BOS within 12 months after lung transplantation. By monitoring the number of neutrophils in BALF, CLAD-BOS can be determined earlier (63). Therefore, interrupting neutrophil-dependent pathways may mitigate tissue damage in BO/BOS. A phase I clinical trial of alvelestat (n = 7), an inhibitor of neutrophil elastase (NE), has been used in patients with cGVHD-BOS. In this trial, 6 patients had stable disease, while 1 patient had progression in the setting of pneumonia. Moreover, 2 patients had improvement in FEV1 of 9%, and 4 patients experienced improvement in symptoms (64).

### 2.3 Eosinophils

Elevated blood counts of eosinophils, a type of innate immune cell, are closely linked to the severity of pulmonary fibrosis (65). Under the influence of inflammatory mediators, activated eosinophils induce fibroblasts to produce IL-6 and other pro-fibrotic cytokines (66). Through autocrine or paracrine mechanisms, these cytokines cause the fibroblasts to proliferate and differentiate into myofibroblasts. This results in excessive deposition of extracellular matrix (ECM) within the lung parenchyma, ultimately leading to pulmonary fibrosis. In patients with CLAD-BOS, eosinophil counts also rise, potentially serving as effector cells to exacerbate disease symptoms (67). Using a cut-off of ≥2% eosinophils, a BALF eosinophilia was recorded in patients with CLAD-BOS (25/79; 31.6%), compared to control lung transplant patients without BOS (41/277; 14.8%).

Montelukast is a potent, specific cysteinyl leukotriene receptor antagonist which can significantly decreases blood eosinophils (68). Suguru et al. (69) found that eosinophilia after allogeneic HSCT occurs before the onset of cGVHD, and early eosinophilia may predict the occurrence of cGVHD in children. A phase II clinical trial was shown that montelukast stabilized FEV1% predicted in all patients with cGVHD-BOS (n=23/23, 100%) at six months, improved compared to historical controls (60%) (70).

### 2.4 Natural killer cells

NK cells coordinate both innate and adaptive immune responses by stimulating the maturation of dendritic-like cells and B cells, the polarization of helper T cells, and the activation of cytokines in T cells (71). Notably, NK cells can exert pro-fibrotic (72) and anti-fibrotic (65, 73) effects and may play, through the production of inflammatory mediators, a crucial role in transplant rejection (74). In patients with CLAD-BOS, the number of NK cells in peripheral blood and BALF increases (75–77). Evidence indicates that activated NK cells can exacerbate the severity of CLAD-BOS, and were associated with long-term graft dysfunction and decreased CLAD-free survival (76). In this regard, NK cells were shown to exert harmful effects by interacting with donor-specific antibodies (78, 79), and to exacerbate airway obstruction in allogeneic transplants (80). In patients with CLAD-BOS, there was an increase in NK cells and cytotoxic molecules in NK cell derived exosomes. Injection of anti-NKG2D blocking antibody can alleviate the development of BO in allografted mice by inhibiting the activation of NK cells (81). Controversially, NK cells can also promote tolerance in solid organ allografts by killing donor antigen-presenting cells (APC), which correlates with improved survival (82). In turn, it was reported that NK cells can alleviate the severity of cGVHD-BOS by inhibiting the proliferation of transplanted T cells (83). Due to the important role of NK cells in cGVHD, some drugs are being developed with the intent of exploiting the above mechanisms (84, 85).

### 2.5 Lymphocytes

The role and function of B and T lymphocytes in BO pathophysiology are complex and controversial, and evidence suggests that their interaction may either promote or inhibit the progression of the disease. B cells function by producing antibodies, which mediate humoral immunity, and cytokines that contribute to immune modulation (86). Moreover, B cells can activate antigen-specific CD4^+^ and CD8^+^ T cells, and present antigens to already activated T cells. Traditionally, T cells are considered key cells involved in transplant rejection (87).

Lymphocyte infiltration, lymphoid follicles, and bronchus-associated lymphoid tissue hyperplasia are often observed in BOS (6). Several drugs with regulatory effects on lymphocyte function are being currently evaluated in clinical trials and have the potential to be applied in the treatment of various types of BOS. It is necessary to understand the mechanism of lymphocytes in BO disease for treatment.

### 2.6 B cells

B cells can function as effector cells and regulatory cells in BOS, and it is important to balance these two roles in disease treatment. B lymphocytes promote CLAD-BOS by producing antibodies and presenting antigens through major histocompatibility complex (MHC) molecules (88). Due to antigen presentation, T cell co-activation, and the production of donor-specific antibodies, B cells are traditionally considered to be an important factor in chronic graft failure (89). B cells play an essential role in hyperacute rejection, which is caused by recognition of donor antigens by preexisting antibodies after organ transplantation (86). Regulatory B (Breg) cells secrete the anti-inflammatory factors IL-10 and TGF-β1, which inhibit the progression of CLAD-BOS (90). Smirnova et al. (91) found that B cells are the main source of local antibody production and a major contributor to CLAD-BOS. An animal study by Texier et al. (92) reported that allografts are infiltrated by a large number of B cells organized in germinal centers. These aggregates are strongly regulated in their IgG alloantibody response, and exhibit an inhibited and regulatory profile.

The accurate identification of biomarkers predictive of BOS can improve the prognosis for patients. Compared with patients without cGVHD, the percentage of CD19^+^CD21^low^ B cells, as well as B cell-activating factor (BAFF) levels and the BAFF/CD19^+^ ratio, are notably elevated in patients with newly diagnosed BOS. Accordingly, an elevated frequency of CD19^+^CD21^low^ B cells was proposed to represent a potential novel biomarker for predicting early risk of BOS in HSCT patients, potentially impacting prognosis (93). B cells play a pivotal role in antigen presentation in autoimmune diseases (94), and their function is particularly evident at low antigen thresholds. Cytokines can trigger an immune response to self-antigens, leading to the development of BO after administering anti-MHC antibodies. Nevertheless, B cells exhibit abnormal survival and maturation in BOS (95).

Srinivasan et al. (96) found that the development of BO in mouse models required the deposition of allogeneic antibodies from donor B cells and the formation of germinal centers. Ibrutinib, a small molecule drug that inhibits the B cell receptor pathway, has been approved by the FDA for the treatment of cGVHD (97). Although entospletinib combined with steroids as frontline treatment for cGVHD was terminated for lack of efficacy, fostamatinib (phase I trial) produced an ORR of 77% and allowed for a strong and durable steroid-sparing effect (98). Meanwhile, phase II clinical trials are currently ongoing to test the clinical efficacy of rituximab (the clinical response rate was 70%) (99) and ofatumumab (the ORR was 62.5%) (100), two monoclonal antibodies which are specific anti–B-cell therapy and beneficial for patients with steroidrefractory cGVHD.

### 2.7 CD8^+^ T cells

The primary function of CD8^+^ T lymphocytes is to identify endogenous antigens, directly kill intracellular and extracellular pathogens, and eliminate infected, mutated, and cancerous cells (101). After lung transplantation, CD8^+^ T cells exhibit high cytotoxic activity and pro-inflammatory properties. A study described the presence of inflammatory infiltration of bronchioles in lung biopsies of 23 children with BO, and compared it with the infiltration of histologically normal airways (102). It was found that CD3^+^ T cells were the most common cell type in BO patients, represented mainly by the CD8^+^ T cell subtype. In addition, lung histopathology showed that CD4^+^ and CD8^+^ T cells were also significantly increased in animal models of BO (103).

BOS is associated with steroid resistance, down-regulation of CD28 expression in pro-inflammatory CD8^+^T cells in peripheral blood, and upregulation of selective costimulatory molecules (104). Increased abundance of cytotoxic/pro-inflammatory CD8^+^T cells, in association with exacerbated fibrosis, has been demonstrated in small distal airways in CLAD-BOS (105, 106). Khatri et al. (107) found that cytotoxic CD8^+^T cells not only accumulate in CLAD-BOS lungs, but also lead to targeted basal cell death in early CLAD-BOS airways. These findings suggest that infiltrating CD8^+^ T cells contribute importantly to the progression of BOS.

Patients with CLAD-BOS may exhibit relative resistance to immunosuppressive agents, including glucocorticoids (GC), which limits the effectiveness of immunosuppressive therapy to prevent pro-inflammatory cytokine secretion (108). Specifically, the stimulation and activation of CD8^+^ T cells during the disease process further accentuates this resistance, which may be associated with reduced GC receptor (GCR) expression in pro-inflammatory CD8^+^ T cells. Decrease expression of inhibiting p-glycoprotein-1 in GC-resistant T-cells would be further increased GCR in patients with CLAD-BOS. Therefore, upregulating GCR expression in CD8^+^ T cells may improve the prognosis of CLAD-BOS. T cells can induce epithelial cell apoptosis through the secretion of granzyme B, and inhibiting this enzyme can reduce the incidence of CLAD-BOS (18). Also, the upregulation of the gene encoding granzyme A in CD8^+^ T cells during homing to lymphoid tissues is linked to the activation of the non-canonical NF-κB pathway (95). However, gabexate mesylate, a synthetic serine protease inhibitor, and methylprednisolone, a synthetic GC, showed no impact on the production of granzyme B in CD8^+^ T cells *in vitro*, while tacrolimus and cyclosporine A, which inhibit IL-2 production, exhibited only modest effects (109). These findings indicate a clear need to identify effective T cell-targeted immunosuppressants for the prevention and treatment of CLAD-BOS (110).

### 2.8 Th1/Th2 cells

In 1986, Mosmann et al. identified two different types of CD4^+^ Th cells (later defined as Th1 and Th2) that exhibited distinct cytokine profiles (111). One theory of immune modulation posits that Th1 and Th2 cells operate between states of stability (112), directing distinct immune response pathways through the production of cytokines. Th1 cells produce interferon (IFN)-, IL-2 and tumor necrosis factor (TNF)-, which cause phagocyte dependent inflammation. Th2 cells produce IL-4, IL-5, IL-6, IL-9, IL-10 and IL-13, which cause strong antibody response and inhibit the functions of phagocytes (113). Specifically, Th1 cells can stimulate macrophages through TNF and IFN, driving cellular immunity against viruses and other pathogens. It can also trigger delayed type hypersensitivity skin reactions. Th2 cells drive in turn humoral immunity, by inducing the production of antibodies by B cells to combat pathogens. While in healthy subjects the balance between Th1 and Th2 is in a state of dynamic equilibrium, alterations in either pathway can influence the other, and overactivation of either pathway can lead to the onset and progression of diseases. Indeed, research has shown that the Th1/Th2 balance plays a pivotal role in the progression of pulmonary diseases (114–116).

Lymphocytes infiltrating in BOS-affected lungs release a variety of inflammatory cytokines, especially Th1 cytokines (105, 117, 118). Th1 cytokines are significantly elevated in peripheral blood, BALF, and lung tissue of patients with BO/BOS (103, 109, 119–121). However, studies have shown that there was no significant difference in the levels of IFN-γ, IL-4, and IL-10 between the peripheral blood of children with PIBO and that of healthy controls, suggesting that Th1/Th2 imbalances may not be related to PIBO pathophysiology (122). In turn, whether Th2 cells mediate pro-fibrotic or anti-fibrotic effects in BO is controversial. Th2 cells can accelerate rejection by releasing pro-inflammatory and potent pro-fibrotic mediators such as IL-4, IL-6, and IL-13 (39, 123, 124). However, by releasing IL-10, Th2 cells can reduce the severity of cGVHD in animal models (125). These findings suggest that Th2 cells have different effects on BOS and fibrosis in different pathophysiological contexts.

A Phase II clinical trial (n =10) to assess aldesleukin, a recombinant analog of IL-2, was conducted for treatment of steroid-refractory cGVHD. The response rate was 80% when assessed by intent to treat in this trial (126). Besides, a potential Th2-targeted therapy is represented by romilkimab, a bispecific IL-4/IL-13 neutralizing antibody that is being evaluated in a phase II clinical trial as anti-fibrosis treatment in systemic sclerosis (127). Romilkimab (n = 47) resulted in a statistically significant decrease in modified Rodnan skin score (mRSS) from baseline to week 24 versus placebo (n = 48) in this trial. However, more research is needed to develop drugs targeting Th1-dependent inflammation, as TNF-α blockade failed to improve small airway obstruction in rheumatoid arthritis patients (128).

### 2.9 Th17 cells

The process by which CD4^+^ T cells differentiate into Th cells is influenced by specific cytokines and costimulatory molecules acting on the T cell receptor (TCR) (129). Th17 cells, a subset of effector Th cells, secrete cytokines and exert effects that are distinct from those of Th1 and Th2 cells (130). Evidence shows that Th17 cells primarily contribute to the onset of various autoimmune and inflammatory diseases via secretion of IL-17, which acts on a variety of cells, including endothelial, mesenchymal, epithelial, and hematopoietic cells (131). Through IL-17-related mechanisms, Th17 cells participate in immune regulation and pathophysiological changes of lung diseases such as COPD, asthma, pulmonary fibrosis, pulmonary arterial hypertension, and lung cancer, among others (132).

During the development of CLAD-BOS, Th cells are induced to differentiate into immunomodulatory Th17 cells (133). After lung transplantation, IL-17 mRNA levels in the BALF of patients with CLAD-BOS were shown to be significantly increased compared to clinically stable lung transplant recipients. In mouse models, there is a correlation between IL-6 and IL-17 levels and tracheal obstruction, and blocking IL-6 can mitigate allograft fibrosis by decreasing the IL-17 transcripts (134). Th17 cells may indeed represent key regulators of airway fibrosis, as inhibiting the function of Th17 cells was shown to reduce the severity of airway fibrosis in BO (103, 135).

Th17 cells play also a deleterious role in cGVHD. Forcade et al. (136) showed that belumosudil, a rho-associated coiled-coil kinase 2 (ROCK2) inhibitor, decreases both severity of murine BO and clinical scores in sclerodermatous cGVHD through inhibition of STAT3 and activation of STAT5. Besides, belumosudil was associated with a best ORR of 32% for patients with early stages of cGVHD-BOS (137). In 2021, the FDA approved belumosudil for the treatment of cGVHD after failure of at least two prior lines of systemic therapy (97).

### 2.10 Regulatory T cells

Treg cells (Tregs) are specialized CD4^+^ T cells typically defined by expression of FOXP3 and CD25 (IL-2 receptor alpha chain) (138). The inhibitory functions of Tregs are crucial to limit inflammation, maintain peripheral tolerance, and curtail the development of autoimmune and autoinflammatory diseases, allergies, acute and chronic infections, cancer, and metabolic inflammation (139). Ample evidence supports the involvement of Tregs in the regulation of immune cell interactions in lung diseases, including parasitic infections, pneumonia, COPD, asthma, fibrosis, and lung cancer (90, 140).

Tregs may enhance EMT and contribute to the progression of pulmonary fibrosis by secreting cytokines such as PDGF and TGF-β, or curb its progression by facilitating the repair of damaged epithelial cells, suppressing fibroblast accumulation, and inhibiting the production and activity of pro-inflammatory factors and cells (141).

However, the role of Tregs in lung fibrosis is controversial, as they were shown to alternatively promote and inhibit fibrosis by enhancing inflammation and contributing to tissue repair, respectively (142, 143). The balance between Tregs and Th17 cells is implicated in pulmonary fibrosis. Th17 mediate autoimmune responses and inflammation, whereas Tregs suppress inflammation and ensure immune homeostasis (144). It was reported that within 3 years after lung transplantation, the proportion of circulating Tregs was significantly higher in patients with BOS than in patients with non-BOS. The risk of BOS in patients with increased proportion of Tregs after transplantation was 2 times higher than that in patients without increased proportion of Tregs (145). Likewise, BO was more severe in animal models implanted with peripheral blood mononuclear cells depleted of CD4^+^ CD25^high^ cells, which suggests that Tregs may have a protective effect on BOS. Accordingly, studies revealed that Tregs can reduce airway fibrosis in mice that developed BO after heterotopic bronchial transplantation (146, 147). However, other studies suggested that Tregs may exert instead pro-fibrotic roles. In mice with ischemic cardiomyopathy, Tregs ablation alleviated hypertrophy and myocardial fibrosis (148). Similarly, in renal fibrosis animal models, inhibiting Tregs differentiation was shown to reduce fibrosis (149). These findings suggest that Tregs have different effects on fibrosis in different pathophysiological contexts.

Extensive efforts are made to develop therapies targeting Tregs. Ruxolitinib, a JAK-STAT pathway inhibitor, can decrease collagen deposition and improve lung function in a mouse model of cGVHD by reducing the polarization of CD4^+^ T cells towards IFN-γ and IL-17A-producing cells and increasing their conversion to Tregs (150). A phase II multicenter trial of ruxolitinib to treat cGVHD-BOS showed that newly diagnosed BOS experienced more dynamic early changes in FEV1 while established BOS was stable, comparing PFTs at baseline. The best lung-specific ORR for the entire study is 34% (151). In 2021, the FDA approved ruxolitinib for the treatment of cGVHD after failure of one or two lines of systemic therapy (97).

## 3 Conclusion

Studies on patients and animal models indicate that immune dysregulation contributes to the onset and progression of airway obstruction and fibrosis in BO. Immune cells interact with each other and influence pathophysiological changes by secreting cytokines to regulate effector cells. In different BO subtypes, macrophages, neutrophils, eosinophils, Th1, and Th17 cells may contribute to disease progression, while Th2, NK, and Tregs seem to have controversial roles (**Figure 1**). cGVHD-BOS is treated with steroids – with ruxolitinib, belumosidil, and now potentially axatilimab for steroid refractory disease. In addition, systemic steroids, fluticasone-azithromycin-montelukast and inhaled long-acting bronchodilator are first-line treatment options for CLAD-BOS. However, none of these drugs are likely to reverse advanced fibrosis/severe obstruction. As research progresses on the complex interplay between immune cells shaping BO development, immune therapy and targeted immune modulators may emerge.
